# Innovation across 13 ungulate species: problem solvers are less integrated in the social group and less neophobic

**DOI:** 10.1098/rspb.2022.2384

**Published:** 2023-04-12

**Authors:** Alvaro L. Caicoya, Alina Schaffer, Ruben Holland, Lorenzo von Fersen, Montserrat Colell, Federica Amici

**Affiliations:** ^1^ Department of Clinical Psychology and Psychobiology, Faculty of Psychology, University of Barcelona, 08007 Barcelona, Spain; ^2^ Institute of Neurosciences, University of Barcelona, 08021 Barcelona, Spain; ^3^ Behavioral Ecology Research Group, Institute of Biology, University of Leipzig, 04103 Leipzig, Germany; ^4^ Department of Human Behavior, Ecology and Culture, Max Planck Institute for Evolutionary Anthropology, 04103 Leipzig, Germany; ^5^ Zoo Leipzig, 04015 Leipzig, Germany; ^6^ Nuremberg Zoo, 90480 Nuremberg, Germany; ^7^ Research Group Human Biology and Primate Cognition, Institute of Biology, University of Leipzig, 04103 Leipzig, Germany; ^8^ Department of Comparative Cultural Psychology, Max Planck Institute for Evolutionary Anthropology, 04103 Leipzig, Germany

**Keywords:** innovation, problem solving, ungulates, neophobia, fission–fusion, social integration

## Abstract

Innovation is the ability to solve new problems or find novel solutions to familiar problems, and it is known to provide animals with crucial fitness benefits. Although this ability has been extensively studied in some taxa, the factors that predict innovation within and across species are still largely unclear. In this study, we used a novel foraging task to test 111 individuals belonging to 13 ungulate species—a still understudied taxon. To solve the task, individuals had to open transparent and opaque cups with food rewards, by removing their cover. We assessed whether individual factors (neophobia, social integration, sex, age, rank) and socio-ecological factors (dietary breadth, fission–fusion dynamics, domestication, group size) predicted participation and performance in the task. Using a phylogenetic approach, we showed that success was higher for less neophobic and socially less integrated individuals. Moreover, less neophobic individuals, individuals of domesticated species and having higher fission–fusion dynamics were more likely to participate in the task. These results are in line with recent literature suggesting a central role of sociality and personality traits to successfully deal with novel challenges, and confirm ungulates as a promising taxon to test evolutionary theories with a comparative approach.

## 1. Introduction

Innovation can be defined as the ability to solve new problems or find novel solutions to familiar problems [1,2]. In humans, innovative behaviour has played a crucial role for the success of our species [3–5]. However, innovation is thought to also provide fitness benefits in species other than humans, especially in complex dynamic environments where socio-ecological challenges often vary [1,2,6–10]. Innovation, for instance, can be highly adaptive to exploit new food sources, to innovatively reduce predation pressure, or to effectively cope with environmental changes by better adapting to novel ecological conditions [1,2,9,11–17]. From great tits (*Parus major*) opening milk bottles [18] to chimpanzees (*Pan troglodytes*) using new tools to solve novel foraging problems [19], experimental evidence has clearly shown that innovation is widespread in the animal kingdom [3,13,20,21]. To date, however, it is still unclear which factors predict the distribution of innovation across species and individuals [22].

At the species level, several studies have assessed the link between the ability to innovate and specific socio-ecological characteristics of the species (e.g. [23–29]). In birds, for instance, species that exploit a larger variety of habitats [23,30] or have a more urbanized lifestyle [24] also show a higher innovation rate. Similarly, the frequency of proto-tool use predicts innovation rate in primates [25] and birds [26], whereas group living is linked to the innovative exploitation of novel food sources in both birds ([28,29], but see [31]) and fish [27]. To date, the largest majority of comparative studies on innovation have been conducted in birds and primates, with few exceptions (fish: [11]; carnivores: [32]; meerkats: [33]; rodents: [2]). However, different evolutionary pressures may be at play across species, and the inclusion of other taxa is essential to understand the limits and generalizability of specific evolutionary hypotheses (see [34–36]). Some species, for instance, show high levels of fission–fusion dynamics (i.e. individuals frequently split into subgroups of varying size and composition) and may require higher cognitive skills (e.g. analogical skills, inhibition) to deal with such complex sociality [37–41]. Similarly, complex cognitive skills may be linked to a wider dietary breadth to facilitate the recognition and processing of different food types [42–44], or to social group size to allow individuals to more effectively deal with a high number of different social partners [45–47]. Fission–fusion dynamics, dietary breadth and social group size may therefore be linked to higher cognitive skills and better innovation skills. Finally, domestication might also affect the ability to innovate, as domesticated species have been selected for traits and skills that facilitate interactions with humans, and may thus be more likely to interact with anthropogenic stimuli and innovate [48,49]. For some authors, however, it is also possible that domestication reduces the ecological challenges that individuals in these species face [50–52], leading to an overall reduction of cognitive skills and brain size [53].

At the individual level, innovation has been linked to an excess of energy, and it is thought to be more common in individuals that have a higher daily food intake and can thus devote more time and/or energy to innovation [20]. More recent work, however, suggests that innovative behaviour, by entailing important risks, may be more common in individuals that have more limited access to resources and need to rely on innovative behaviour to survive [2,11,54]. Therefore, innovation should be more common in lower-ranking individuals, who usually have little access to resources, but also in females and younger individuals, as they generally have higher metabolic costs (for a review, see [22]). Moreover, other factors may explain inter-individual variation in innovation. Neophobia, for instance, is the fearful reaction to novel stimuli or situations [55], and might decrease the likelihood that individuals take part in novel tasks and solve novel problems [12,56,57]. However, although little neophobia is likely to facilitate interaction with novel set-ups, its link to innovation is still debated [22,58]. Moreover, social integration may also explain inter-individual variation in innovation. Across taxa, social integration is known to provide crucial fitness benefits to individuals (primates: [59,60]; humans: [61,62]; horses: [63]) and this may affect the potential payoffs when responding to novelty, with more integrated individuals being less likely to interact with novel stimuli or situations than less integrated ones (see [57]).

In this study, we aimed to assess the factors that predict interspecific and intra-specific variation in ungulate innovation. Ungulates offer an exceptional model for comparative research as they show a remarkable variety of socio-ecological characteristics, including differences in fission–fusion dynamics, dietary breadth and sociality (from non-social to monogamous and large mixed stable groups) [41,57,64–67]. Moreover, ungulates show important variation in terms of cognitive skills [57,66], which might be linked to the socio-ecological variation that characterizes them. Finally, ungulates have already shown complex problem solving skills in novel foraging tasks [68], making them an ideal candidate taxon for the study of interspecific and intra-specific variation in innovation. Here, we tested 111 individuals of 13 ungulate species by providing them with novel transparent and opaque cups, which they had to open to retrieve food. We then assessed whether innovation (i.e. participating and solving the task, latency to solve the task, variety of behaviours used for this purpose) differed across species and subjects depending on their socio-ecological and individual characteristics. Based on existing literature, we focused on the following socio-ecological traits, which might be linked to higher cognitive skills and/or greater ability to innovate: fission–fusion dynamics [37–40], dietary breadth [42–44], social group size [45–47] and domestication [48,49]. We therefore predicted that innovation should be more likely in species with higher fission–fusion dynamics (Prediction 1), with a wider dietary breadth (Prediction 2), living in larger groups (Prediction 3) and/or having been domesticated (Prediction 4). In terms of inter-individual variation, we followed literature suggesting that innovative behaviour should be more common in individuals who have more limited access to resources [2,11,54], in those who react more positively to novelty and in those who are less integrated in their social group (see 57). We therefore predicted that innovation should be more likely in more subordinate individuals (Prediction 5), in females (Prediction 6), in younger individuals (Prediction 7), in less neophobic ones (Prediction 8) and in individuals that are less integrated in the social group (Prediction 9).

## 2. Methods

### (a) Subjects

We studied 111 subjects belonging to 13 ungulate species, including six impalas (*Aepyceros melampus petersi*), 13 mhorr gazelles (*Nanger dama mhorr*), 13 dorcas gazelles (*Gazella dorcas osiris*), seven scimitar oryx (*Oryx dammah*), seven dromedaries (*Camelus dromedarius*), seven red deer (*Cervus elaphus*), 15 Barbary sheep (*Ammotragus lervia*), six giraffes (*Giraffa camelopardalis rothschildi*), four guanacos (*Lama guanicoe*), four lamas (*Lama glama*), four Przewalski horses (*Equus ferus przewalskii*), nine sheep (*Ovis aries*) and two groups of goats (*Capra aegagrus hircus*), one with nine and one with seven individuals. All subjects were housed with conspecifics of different sex and age at the zoos of Barcelona, Barben, Nuremberg and Leipzig, and were all individually recognizable. None of the study subjects had ever been tested in an innovation test before, although all species occasionally participated in enrichment activities, and three of the six giraffes had previously participated in other cognitive tasks [69,70]. Based on existing literature, we further classified the study species according to their socio-ecological characteristics, including dietary breadth, the presence of fission–fusion dynamics and domestication (for more details on the study subject and the species classification, see electronic supplementary material).

### (b) Behavioural observations

In each study group, we conducted behavioural observations to assess individuals' dominance rank and social integration in the group. First, we assessed dominance hierarchy by using *all occurrence sampling* to record all dyadic agonistic interactions in each group, with a clear winner–loser outcome (i.e. threat, chase, fight). We then used the Elo method [71] with the EloRating package (v. 3.5.0; [69]), setting 1000 as the individual start values and 100 as the *k* factor—a weighted constant based on winning probability [72,73]. Finally, we averaged these values through the study period, and standardized them to range from 0 (i.e. lowest rank) to 1 (i.e. highest rank). For 21 individuals (three dorcas gazelles, six giraffes, two goats, one impala, five mhorr antelopes, one scimitar oryx, two red deer, one sheep) we observed no agonistic interactions throughout the study period, and their rank was therefore assessed by the experimenter together with the animal keepers, based on observations of priority of access to food [57]. Second, we assessed Eigenvector centrality as a measure of individual social integration. We assessed spatial proximity networks in each study group by conducting 100 *instantaneous scans* per group. Scans were conducted every 15 min across several days, recording the spatially closest individual (nearest neighbour) of each group member [73]. We then built an undirected weighted matrix for social network analyses and used the vegan (v. 2.5-3; [74]), asnipe (v. 1.1.10; [75]) and igraph packages (v. 1.2.1; [76]) in R to assess individuals' Eigenvector centrality (i.e. a measure proportional to the sum of the centralities of each individual's neighbours, which assesses the importance of individuals as ‘social hubs'; [77,78]). As we had no social network data for seven individuals (two goats, one impala, three mhorr antelopes and one sheep), we conventionally assigned them the average centrality value for that study group [57]. To ensure that this conventional attribution of centrality did not bias our results, we also repeated all the analyses after removing these seven individuals, and found identical results for all the models (see below).

### (c) Neophobia

All of our study subjects were previously tested with a neophobia task in which individuals were exposed to familiar food, part of which was positioned close to a novel object [57]. We used these data to calculate a neophobia index, as the proportion of time in which individuals approached the side with no object, out of the total time they spent in proximity of the food from either side. More detailed analyses on neophobia for most individuals (*N* = 78) have been already published [57]. Out of the 111 study subjects, 15 individuals (one barbary sheep, four dorcas gazelles, one giraffe, four goats, one scimitar oryx, one Przewalski horse, one red deer and two sheep) did not participate in the task when the novel object was present. If they participated in previous sessions where no novel object was present (*N* = 8; one giraffe, four goats, one scimitar oryx, one Przewalski horse and one red deer), we assumed that it was the presence of the object that prevented them from participating, and assigned them the highest possible score for neophobia (i.e. [1]). We assigned a neutral value of 0.5 to all the individuals that did not participate either in the presence or in the absence of the novel object (*N* = 7), as the presence of the novel object had no effect on their behaviour in the task.

### (d) Innovation task

**Figure 1.**
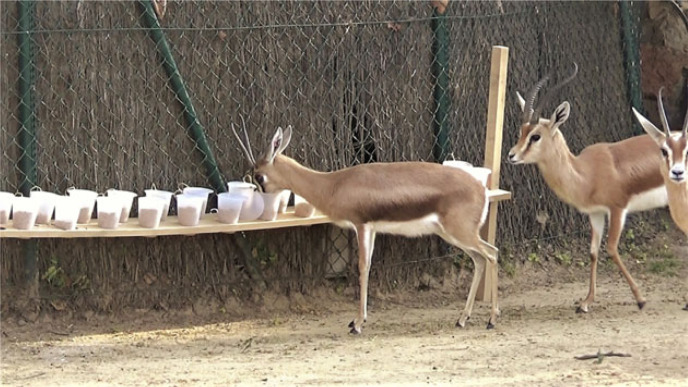
A dorcas gazelle retrieving food after removing the cover from a cup.

We tested all study groups with an innovation task. The task was conducted in a familiar environment (i.e. the external enclosures) when all group members were present. During the task, we presented the group with identical plastic cups, which had an opaque cover on top and were inserted on a long rigid board. All cups were filled with a highly favourite food reward (i.e. carrots, alfalfa, fodder or food pellets, depending on the species), which could be reached with the muzzle after removing the cover. The number of cups was proportional to the study subjects in the group. The board was positioned in an area of the enclosure often used by the study groups. A session started when the board was in place and the experimenter left the enclosure, and lasted up to 20 min or until all the food was gone. All study groups received two sessions, on two different days: a first session with transparent cups (i.e. transparent condition), in which food was visible, and a second session with completely opaque cups (i.e. opaque condition), in which food was not visible. Impalas, however, only received the first session because the COVID pandemics prevented us from finishing the task, and the group composition had changed when testing was again possible. We video-recorded all sessions and we later coded from the videos: (i) whether subjects participated in the task (i.e. whether they approached with the muzzle within 1 m from the cups), (ii) whether they solved the task (i.e. whether they successfully opened the cup and retrieved the food), (iii) the individual latency to solve the task for the first time (i.e. the total amount of time spent in proximity to the cups before first opening one), (iv) the strategy used to open each cup (e.g. opening the lid with the lips, nose, muzzle or tongue) and (v) the exact duration of the session (see figure 1 for a picture of the set-up).

### (e) Statistical analyses

We used the MCMCglmm package (v. 1.0.1; [79]) in R (v. 3.5.0; [79]) to run generalized linear mixed models [80] with phylogenetic controls. To control for phylogenetic relationships across study species, we prepared a consensus tree with the package ape [81], based on 10 000 trees that we subsampled and pruned from the mammal tree of life to match the species included in our study [82]. From the consensus tree, we obtained a covariance matrix with the phylogenetic relationship between species, which we then included in the models (for a similar approach, see e.g. [57,83–85]).

We conducted three sets of models, to assess whether participation (as binomial dependent variable: Model 1), success (as binomial dependent variable: Model 2) and probability of using more than one strategy to solve the task (as binomial dependent variable: Model 3) varied across species and individuals. In Models 1 and 2, we included a line for each study subject and condition, whereas in Model 3, we only included individuals that solved the task. In all the models, we included as test predictors whether the species has fission–fusion dynamics, whether it is domesticated, whether it has low or high dietary breadth, and the group size of the study groups. As test predictors, we also included the individuals' sex, age, rank, Eigenvector centrality and neophobia index, as defined above. Finally, in the first two models, we controlled for condition (i.e. transparent or opaque) and session duration, including subject identity as random factor, whereas in the last model we only controlled for the overall number of trials solved by each individual.

We then used AIC values to compare each of the three models above to an identical model including phylogenetic controls, controls and random factors, but no test predictors. If this comparison suggested that the more complex model provided a better fit to the data, we assessed the posterior mean, 95% credible intervals (CIs) and pMCMC of the single test predictors. Terms with pMCMC values lower than 0.05 were considered statistically significant (see e.g. [85]). All models included non-informative priors, 1 000 000 iterations, a burn-in of 100 000 and a thinning interval of 300 to minimize autocorrelation and facilitate convergence (see [85,86]). We repeated the analyses three times, and visual inspection of the models suggested no convergence issues (data and script are available in the electronic supplementary material).

## 3. Results

On average, 62% of the study subjects participated in at least one condition of the task. However, participation varied widely across species, with 100% of the dromedaries approaching the cups but only 33% of the sheep. Overall, only 36% of the study subjects were successful in retrieving food at least once. The species with a higher percentage of successful individuals were dromedaries and goats, with 86% and 69% of the individuals opening the cups, respectively. Among the individuals that solved the task, latency to open the cup for the first time was on average 51 s, ranging from an average of 6 s for Prewalski horses to more than 5 min for mhorr gazelles. Finally, we found that only nine out of 40 successful individuals used more than one strategy to solve the task, including 3 of the 6 successful dromedaries and both successful scimitar oryx.

After accounting for phylogeny, the more complex model for Model 1 provided a better fit to the data than the simpler one (complex model, AIC: 124.8, weight: 0.993; simple model, AIC: 134.7, weight: 0.007). Participation was higher in species with fission–fusion dynamics (posterior estimate: 7.2 [95% CIs: 0.5 to 14.5], *p* = 0.010), in domesticated species (posterior estimate: 6.7 [95% CIs: 0.9 to 13.7], *p* = 0.005) and in individuals with lower neophobia (posterior estimate: −12.8 [95% CIs: −24.5 to −3.3], *p* = 0.001). For Model 2, the more complex model provided a better fit to the data than the simpler one (complex model, AIC: 110.4, weight: 0.871; simple model, AIC: 114.2, weight: 0.129). The probability of success was predicted by lower levels of neophobia (posterior estimate: −23.0 [95% CIs: −41.2 to −7.1], *p* < 0.001) and by lower integration in the social network (posterior estimate: −13.4 [95% CIs: −32.7 to 1.8], *p* = 0.047). Finally, the simpler model provided a better fit to the data than the more complex one for Model 3 (simple model, AIC: 13.0, weight: 1; complex model, AIC: 28.7, weight: 0), suggesting that none of the test predictors we included reliably predicted inter-individual and interspecific variation in the probability of using more than one strategy to solve the task.

## 4. Discussion

Our study showed interspecific and intra-specific variation in innovation, in our study sample. In particular, we found differences in the probability that ungulates participated in the task and solved it. Domesticated species and species with higher fission–fusion dynamics were more likely to participate in the task, and so were individuals that were less neophobic to novel objects. Moreover, less neophobic individuals and socially less integrated ones were more likely to solve the task. By contrast, we found no differences across individuals or species in the latency to solve the task or in the probability of using more than one strategy to retrieve food (see electronic supplementary material for a video clip with an individual of each species solving the task using different strategies).

Species with higher fission–fusion dynamics and domesticated species were more likely to participate in the task, although they were not better at solving it. Domestication may facilitate interaction with novel set-ups and be linked to an increased interest in anthropogenic objects, as suggested by studies in other taxa (e.g. in captive canids [87] and birds [88]). However, this would not necessarily lead to an increase in problem solving skills, as the domestication process might have specifically selected for traits and skills that facilitate interactions with humans (and human artefacts), but not for cognitive skills that allow more efficient problem solving (e.g. in captive dogs and wolves [89]). Moreover, also species that show higher fission–fusion dynamics in the wild were more likely to participate in the task, but not to solve it. Fission–fusion dynamics have been linked to enhanced cognitive skills, like inhibition and analogical reasoning [41], which may increase behavioural flexibility and problem solving abilities (e.g. in humans [90] and wild birds [91]). However, our study failed to find a link between fission–fusion dynamics and innovation, and there are at least two reasons for that. First, it is possible that fission–fusion dynamics need to be experienced during ontogeny to drive variation in cognitive skills (but see [37] in captive primates). As our study subjects were all captive, this might have prevented us from finding a relationship between the two variables (see below). Second, not all forms of fission–fusion dynamics might be linked to an increase in cognitive skills. Aureli *et al*. [41], for instance, highlighted how the emergence of higher socio-cognitive skills may be limited to some of the different phylogenetic routes by which fission–fusion dynamics evolved. In particular, when fission–fusion dynamics evolve in group-living species, individuals already rely on a set of complex skills that are necessary for living in a group, and they might thus evolve more complex ones—something that would not be possible when fission–fusion dynamics emerge in solitary species [41]. Therefore, more detailed ecological data will be necessary to better quantify fission–fusion dynamics in ungulates and identify the phylogenetic routes by which they emerged.

Dietary breadth failed to significantly predict interspecific variation in innovation. These results are in contrast with other studies, which have shown a significant link between dietary breadth and cognitive skills (e.g. in captive and wild birds: [92,93]; in wild primates: [43,94]). These differences may be explained in at least three ways. First, it is possible that different evolutionary pressures are at play across different taxa. Therefore, whereas dietary breadth might play a crucial role in the emergence of complex cognitive skills in birds or primates [43,94], other socio-ecological characteristics may be more relevant in ungulates for the emergence of problem solving skills. Moreover, it is possible that our limited sample size, which only included captive individuals, did not allow us to detect interspecific variation because sample size was too small and captive individuals may not be representative of their wild counterparts (see below for a better discussion). Finally, it is possible that our current socio-ecological classification should be improved by the inclusion of more precise socio-ecological data, because it is currently based on studies that used very different methods (see below).

In terms of intra-specific variation, less neophobic and socially less integrated individuals were more likely to solve the innovation task. Individuals with lower neophobia were indeed more likely to participate and successfully open the cups. Little neophobia may facilitate interaction with novel set-ups, without necessarily being linked to higher innovation [58]. However, when the set-up is relatively easy and does not require subjects to have a complex understanding of the contingencies of the task, as in our study, non-causal manipulation of the set-up might be sufficient to solve the task. Future studies should therefore ideally test how performance changes with more complex tasks. Our results are also in line with other studies showing a link between higher innovation rate and lower neophobia in wild [12,23,56,95–98] and captive animals [12,56,99–101]. Moreover, our study showed that little integration in the social network was linked to higher innovation. These findings provide support for the hypothesis that, also in ungulates, socially less integrated individuals may be more likely to interact with novelty and to innovate (see example in captive ungulates [57]). Less integrated individuals may more likely overcome neophobia and deal with novel socio-ecological challenges to get a better share of resources, likely because they have to overcome the lower fitness benefits of low social integration (in humans, see: [61,62], in wild primates [59,60,102–104]) and/or because their social position does not allow them to adequately rely on social information (see e.g. [105] for a negative relationship between individual innovation and social learning in primates). Our findings are also in line with recent literature in wild [106] and captive [107] primates, showing that socially less integrated individuals are less likely to obtain resources and more likely to overcome neophobia when food is unevenly distributed in the group. Finally, it should be noted that, in this study, we measured social integration in terms of spatial proximity between group members. In ungulates, greater distance from other group members may have direct consequences for individual survival, especially when facing high predatory pressure [108]. Therefore, low social integration may be especially important in this taxon as a predictor of problem solving skills, by posing a real challenge for individual fitness.

No other factors included in the analyses predicted intra-specific variation in innovation (i.e. individual's sex, age, rank). These results are in line with a recent meta-analysis of studies on intra-specific variation in innovation [22], which provides no clear support to evolutionary hypotheses linking innovation to these individual traits, either because they would predict excess of energy [20] or a limited access to resources [2,11,54]. Instead, variation in innovation seems to vary across individuals depending on differences in sociality or in traits related to personality, like neophobia [22].

Finally, we did not find a link between the test predictors included in this study and the probability of using more than one strategy to solve the task. This is in contrast with previous studies showing a link between higher motor flexibility (i.e. using more than one technique to solve the task) and higher innovation rate (in wild birds [109]). However, it is possible that other set-ups allowing more variation in the behavioural strategies used to innovate might evidence different patterns. Here, for instance, most individuals opened the cups by using their nose, muzzle or lips, and only 9 of the 111 study subjects used more than one strategy. Still, some individuals explored alternative behaviours to open the cups, by for instance gently lifting the lid with the lips, or throwing the cups on the floor to retrieve the food.

Current limitations of this study include the fact that we could only test a limited number of subjects for each species, and that we only included captive individuals, which may not be representative of their wild counterparts. Socio-ecological constraints experienced during ontogeny [110], continuous exposure to human cultural milieu [111,112], reduced predation risk, high food availability and extensive exposure to novel objects may affect the development of cognitive skills in captive individuals [113], and mask potential differences across individuals and species. Moreover, previous studies suggest that captive animals may more likely interact with new objects and solve novel problems than their wild counterparts [12,22]. Therefore, more studies including wild individuals are required before our findings can be generalized. Another important limitation of our study is that we assessed interspecific variation based on socio-ecological characteristics of the study species as described in the literature. However, the studies we used to categorize species often used different methods, were conducted under very different conditions and had different quality. In the future, it will be essential to bring together experts of ungulate socio-ecology to make a more quantitative categorization of species according to their socio-ecological traits. Finally, we could not include brain size measures as test predictors in our models, because there are no data in the literature for all the species we included. Future studies should ideally target species for which these measures are available, to assess whether different brain size measures and innovation rate are linked in ungulates, as it also happens in other taxa [13,32,114].

Overall, we showed that personality traits and social integration play an important role in ungulates, by reliably explaining variation in problem solving skills. These results are only partially in line with findings in other species, and despite important limitations in our study, they suggest that different evolutionary pressures may be at work in different taxa. Therefore, ungulates constitute a valid model for the comparative study of cognition, and the inclusion of still understudied taxa appears a powerful tool to test the limits of current evolutionary hypotheses.

## Data Availability

The data are provided in electronic supplementary material [115].
